# Naringenin: an analgesic and anti-inflammatory citrus flavanone

**DOI:** 10.18632/oncotarget.14084

**Published:** 2016-12-21

**Authors:** Marília F. Manchope, Rubia Casagrande, Waldiceu A. Verri

**Affiliations:** Departamento de Ciências Patológica, CCB, Universidade Estadual de Londrina, Londrina, Brazil

**Keywords:** flavonoids, inflammation, pain, oxidative stress, nitric oxide, Neuroscience

In this editorial, we discuss recent evidence from our research group on the analgesic and anti-inflammatory mechanisms of the flavonoid naringenin (4’,5,7-tryhidroxy-flavanone). Flavonoids are polyphenolic compounds found in human diet [1]. Naringenin belongs to flavanone class of flavonoids, and it is mainly found in citrus fruits including, lemon, orange, tangerine and grapefruit [1–5]. The antioxidant activity is the most recognized effect of flavonoids, which depends, for instance, on hydrogen donation and electron stabilization in the phenolic rings [1]. Naringenin presents therapeutic effect in several models of inflammatory pain [2, 3, 5]. Naringenin inhibits the pain-like behavior induced by inflammatory stimuli such as phenyl-p-benzoquinone, acetic acid, formalin, complete Freund’s adjuvant, capsaicin, carrageenan [2], superoxide anion [3], and LPS [5]. Moreover, naringenin inhibits UVB irradiation-induced skin inflammatory edema, cytokine production, myeloperoxidase activity, matrix metalloproteinase-9 activity, and oxidative stress [4].

**Figure 1:**
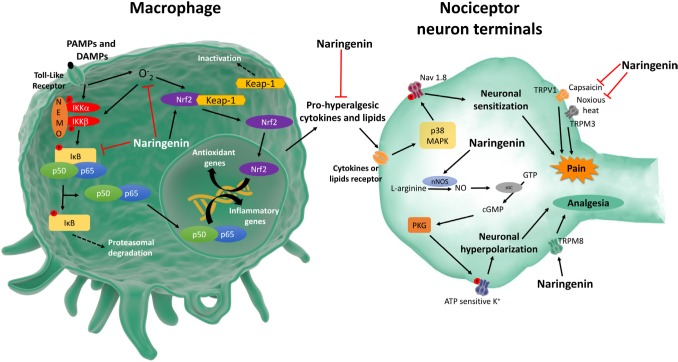
Schematic summary of naringenin analgesic and anti-inflammatory mechanisms

Pathogen (PAMPs) and damage (DAMPs) associated molecular patterns and inflammatory mediators activate resident macrophages. Resident macrophages produce chemotactic molecules to recruit leukocytes to the inflammatory foci, mainly neutrophils in the early events of inflammation. Activated macrophages and neutrophils induce oxidative stress by producing superoxide anion and other reactive oxygen (ROS) and nitrogen species. Naringenin inhibits leukocyte recruitment [2–5] and production of superoxide anion [3–5], whilst increases GSH [2–4], and antioxidant capacity [3–5]. Naringenin also acts on macrophages inducing Nrf2 activation, a nuclear factor that induces antioxidant and anti-inflammatory responses, inducing HO-1 expression [3]. PAMPs, DAMPs and ROS induce NFκB activation in macrophages resulting in the production of pro-hyperalgesic cytokine such as IL-33, TNFα, IL-1β and IL-6. Pro-hyperalgesic cytokines induce the production of lipid mediators such as PGE_2_ that sensitize the nociceptor neurons. Naringenin inhibits LPS- and carrageenan-induced NFκB activation *in vivo* [2] and *in vitro* [5], which contributes to naringenin inhibition of IL-33 [2], TNFα [3–5], IL-1β [2, 4, 5] and IL-6 [4,5] production and expression of cyclooxygenase-2 mRNA [3] (Figure 1).

The ligand-gated and voltage-gated ion channels are essential to the role of nociceptor neurons to sense noxious stimuli [6]. Inflammatory cells-released pro-hyperalgesic cytokines and lipids that bind to their receptors expressed in nociceptor neuron terminals activating intracellular signaling pathways resulting in the modulation of ion channels activity including Nav1.8, TRPV1, and TRPA1. As a consequence, there is nociceptor neuron activation and/or sensitization to nociceptive stimuli, thus, generating pain [6]. The evidence that naringenin inhibits NFκB activation and induces Nrf2 activation is in line with indirect effects over nociceptor neuron activity since inhibiting NFκB and inducing Nrf2 reduce cytokine production and oxidative stress. Thus, naringenin inhibits the production of nociceptive molecules in non-neuronal cells, which will result in reduced activation of nociceptor neurons. For instance, naringenin inhibits NFκB-dependent TNFα and IL-1β production by macrophages [5]. These cytokines induce nociceptive neuron sensitization via p38 MAPK phosphorylation of Nav1.8 sodium channels [6] (Figure 1).

Evidence also support that naringenin directly modulates nociceptor neuron activity. High concentrations of naringenin reduces TRPV1 activation [7], which corroborates the naringenin inhibition of capsaicin-induced overt pain like-behavior and mechanical hyperalgesia [2]. At much lower concentrations compared to TRPV1, naringenin blocks TRPM3 ion channel, a noxious heat sensor channel [7]. Naringenin can also activate TRP channels such as TRPM8, which has been described to induce analgesia [7]. Thus, naringenin regulates TRP channels expressed by nociceptor neurons such as TRPV1, TRPM3 and TRPM8 to induce analgesia (Figure 1).

Nitric oxide (NO) mediates the analgesic effect of opioids, and some non-steroidal anti-inflammatory drugs such as dipyrone [8]. The analgesic effect of NO depends on the induction of the production of second messenger cGMP by activating soluble guanylate cyclase (sGC). Then, the cGMP-dependent protein kinase (PKG) is activated and phosphorylates ATP sensitive K^+^ channel to induce potassium influx hyperpolarizing the nociceptor neurons, thus, inhibiting excitatory nociceptive synaptic transmission [8]. Naringenin inhibits mechanical hyperalgesia [2, 3], thermal hyperalgesia [3] and neutrophil recruitment [2] by activating the NO-cGMP-PKG-ATP sensitive K+ channel signaling pathway (Figure 1) since these effects of naringenin were reduced by the respective inhibitors L-NAME, ODQ, KT5833 and glibenclamide [2, 3].

Concluding, naringenin acts by mechanisms involving the inhibition of leukocyte recruitment [2–5], oxidative stress [2–5], NFκB activation [2, 5] and pro-hyperalgesic cytokine production [2–5] on the immune cells such as macrophages. Nevertheless, naringenin also modulates TRP channels such as TRVP1, TRPM3 and TRPM8 reducing pain [7], and activates a NO signaling pathway that induces nociceptor neuron hyperpolarization [2, 3]. Therefore, naringenin treatment is a promising analgesic, anti-inflammatory and antioxidant compound, requiring further investigation in preclinical models and clinical settings.
